# Survival of Filipino women with breast cancer in the United States

**DOI:** 10.1002/cam4.6403

**Published:** 2023-09-27

**Authors:** David W. Lim, Winston W. Li, Vasily Giannakeas, Tulin D. Cil, Steven A. Narod

**Affiliations:** ^1^ Temerty Faculty of Medicine University of Toronto Toronto Ontario Canada; ^2^ Women's College Research Institute, Women's College Hospital Toronto Ontario Canada; ^3^ Department of Surgery Women's College Hospital Toronto Ontario Canada; ^4^ Division of General Surgery, Department of Surgery University of Toronto Toronto Ontario Canada; ^5^ Dalla Lana School of Public Health University of Toronto Toronto Ontario Canada; ^6^ Division of General Surgery University Health Network (Princess Margaret Cancer Centre) Toronto Ontario Canada; ^7^ Institute of Medical Science, University of Toronto Toronto Ontario Canada

**Keywords:** breast cancer, cancer epidemiology, Filipino

## Abstract

**Background:**

The survival of women with early‐stage breast cancer varies by racial group. Filipino women with breast cancer are an understudied group and are often combined with other Asian groups. We compared clinical presentations and survival rates for Filipino and White women with breast cancer diagnosed in the United States.

**Methods:**

We conducted a retrospective cohort study of women with breast cancer diagnosed between 2004 and 2015 in the SEER18 registries database. We compared crude survival between Filipino and White women. We then calculated adjusted hazard ratios (HR) in a propensity‐matched design using the Cox proportional hazards model.

**Results:**

There were 10,834 Filipino (2.5%) and 414,618 White women (97.5%) with Stage I–IV breast cancer in the SEER database. The mean age at diagnosis was 57.5 years for Filipino women and 60.8 years for White women (*p* < 0.0001). Filipino women had more high‐grade and larger tumors than White women and were more likely to have node‐positive disease. Among women with Stage I–IIIC breast cancer, the crude 10‐year breast cancer‐specific survival rate was 91.0% for Filipino and 88.9% for White women (HR 0.81, 95% CI 0.74–0.88, *p* < 0.01). In a propensity‐matched analysis, the HR was 0.73 (95% CI 0.66–0.81). The survival advantage for Filipino women was present in subgroups defined by age of diagnosis, nodal status, estrogen receptor status, and HER2 receptor status.

**Conclusion:**

In the United States, Filipino women often present with more advanced breast cancers than White women, but experience better breast cancer‐specific survival.

## Graphical abstract

In the United States, Filipino women present with more advanced breast cancers than White women. However, we observed a 27% decreased rate of breast cancer mortality among Filipino compared with White women in a propensity‐matched analysis. The difference in breast cancer–specific survival between Filipino and White women may be a result of inherent biological differences and racial socioeconomic disparities.

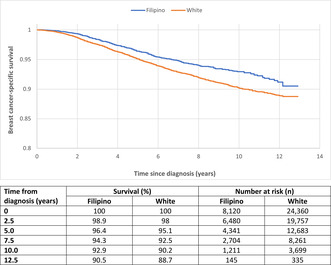

## 1 INTRODUCTION

The Philippines is the thirteenth most populous country in the world with 109 million people and 27,163 new breast cancer cases diagnosed annually.^1^ The annual incidence rate (52.7 per 100,000) in the Philippines is the fourth highest of any Asian country (after Singapore, Japan, and Korea).^2^ In the USA, the annual incidence rate (all women) is much higher (90.3 per 100,000).^3^ In contrast, the annual mortality rate is higher the Philippines than in the USA (19.3 vs. 12.4 per 100,000, respectively).^1^, ^3^ This is the consequence of much poorer survival after breast cancer for women in the Philippines than for women in the United States. The basis for the disparities in incidence, mortality and survival rates relate to inherent risk, screening, access to care or to the intrinsic biology of the cancers.^4^, ^5^, ^6^ Asian women who immigrate to the United States experience higher cancer rates than US‐born Asian Americans.^7^

In the USA, breast cancer survival rates among Black women are lower than those of White women.^8^, ^9^ To a large extent, this is due to the relatively poor survival of Black women with ER‐positive breast cancer,^10^, ^11^, ^12^ but they also have a higher proportion of triple‐negative cancers,^6^, ^12^ and may have less access to screening and care. Less is known regarding survival for other ethnic groups in the USA, including Filipino women. In general, women of Asian ancestry have better survival rates than White women^13^, ^14^, ^15^ but Asian women represent a large and diverse group and all Asian subgroups should not be combined. In the Surveillance, Epidemiology, and End Results (SEER) database, Asians are subdivided into Chinese, Japanese, Vietnamese, Korean, Asian Indian, Pakistani, Filipino, Thai, Laotian, Hmong, and Kampuchean (including Khmer and Cambodian).^16^ Two studies found significant variation in breast cancer survival in the various Asian ethnic subgroups.^17^, ^18^ We recently reported that Chinese women in the United States had significantly better 10‐year breast cancer‐specific survival compared with White women (88.8% vs. 85.6%, HR = 0.71, 95% CI 0.62–0.81).^19^ Emerging literature reveals that there are disparities among Asian American subgroups in accessing treatment, as reflected in wait times to surgery^20^ and radiation.^21^

In Canada, Filipino migrant women with breast cancer present at a relatively young age at diagnosis and at more advanced stage at presentation than White women^22^; whether these differences in breast cancer characteristics impact on survival is unknown. In the current study, we compare survival rates for Filipino and White women with Stage I‐IIIC breast cancer in the SEER database who were diagnosed between 2004 and 2015. We first compared clinical presentation, treatment patterns, and outcomes between the two groups. Second, we conducted a propensity‐matched survival analysis to compare breast‐cancer specific survival rates. This was conducted to see if Filipino ethnicity per se was a risk factor for breast cancer mortality, independent of pathologic factors, presentation and treatments received.

## 2 METHODS

We used SEER*Stat statistical software, version 8.3.6 (National Cancer Institute, Bethesda, MD) to create a case‐listing session. The data that support the findings of this study are available from the National Cancer Institute Surveillance, Epidemiology, and End Results Program. Restrictions apply to the availability of these data, which were used under license for this study. Data are available at https://seer.cancer.gov/data/access.html with the permission of the Surveillance Research Program (SRP) in the National Cancer Institute's Division of Cancer Control and Population Sciences (DCCPS). SEER data covers approximately 35% of the US population.^23^, ^24^ We retrieved records of Filipino and White women diagnosed with a primary invasive breast cancer in the SEER 18 registries database (November 2016 submission). Race data in SEER is determined by a specific algorithm, using all facility resources (e.g., medical records, face sheet, physician and nursing notes, photographs, other sources, etc.), and are subject to repeated quality evaluation and refinement.^16^, ^25^, ^26^ The sources of race data in priority order are: (1) patient's self‐declared identification, (2) documentation in medical records, and (3) death certificate.^16^

**FIGURE 1:**
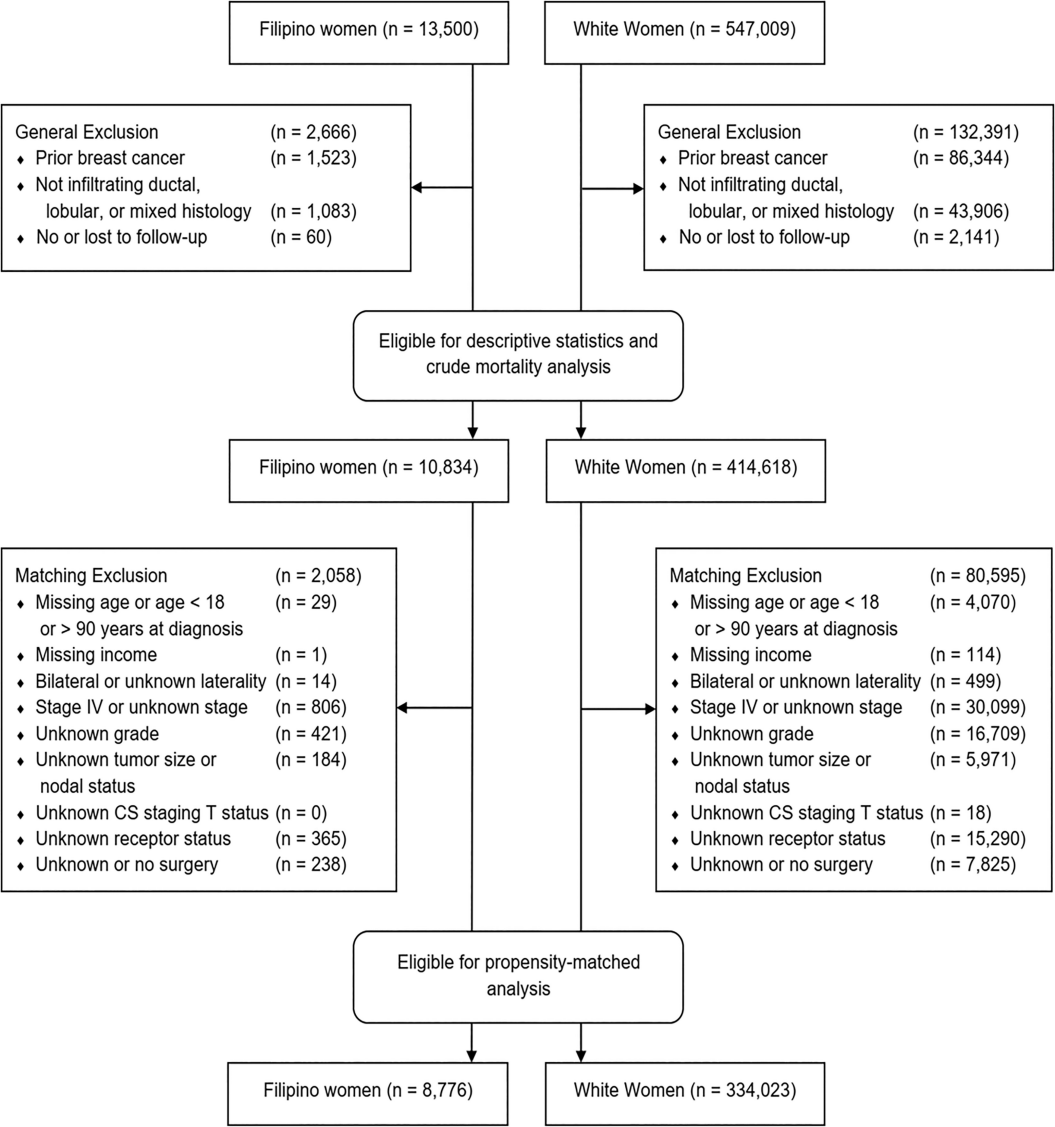
Study flow diagram.

We included women with American Joint Committee on Cancer (AJCC) Stage I‐IV breast cancer diagnosed from 2004 to 2015. We excluded women with a previous history of breast cancer, women with tumors that were not of infiltrating ductal, lobular, or mixed histology, and women who had no follow‐up or who were lost to follow‐up (Figure 1).

This study was exempted from review by the Women's College Hospital research ethics board because patient informed consent was not required. We adhered to the “Strengthening the Reporting of Observational Studies in Epidemiology Statement” guidelines for reporting observational studies.^27^

We collected data on age and year at breast cancer diagnosis, median household income, and marital status. Clinical data included tumor size and grade, laterality, nodal stage, AJCC stage, and estrogen receptor (ER), progesterone receptor (PR), and HER2/neu receptor status. Treatment details included surgical procedure (i.e., lumpectomy, mastectomy), and receipt of chemotherapy and radiotherapy. Information on endocrine therapy was not available in SEER*stat. Vital status included date and cause of death.

### 2.1 Statistical analysis

In the first analysis, Filipino and White women were compared for demographic, pathologic and treatment factors. We compared crude actuarial survival rates for Filipino and White women with Stage I–III breast cancer using the Kaplan–Meier method. Patients were followed from the date of diagnosis until the end of follow‐up, death from breast cancer or another cause, or loss to follow‐up. The log‐rank test was used to compare differences between groups. We calculated the annual mortality rates from breast cancer for each year of follow‐up until 13 years post‐diagnosis. To do this, we considered the number of women alive at the time of the beginning of the interval and the number of breast cancer deaths that occurred during that 1‐year interval.

We then conducted a matched analysis. We compared survival rates for Filipino and White women that were similar for demographic, clinical characteristics and treatment in order to determine if Filipino race per se was an independent prognostic factor. We performed a 1:3 propensity‐matched analysis comparing Filipino to White women with Stage I–IIIC breast cancer. Women were matched on the year and age at diagnosis (both within 2 years), tumor grade, nodal stage, AJCC clinical stage, ER, and HER2 status (positive, negative, or unknown), and propensity score. The propensity score was based on marital status, household income, tumor size, PR status, and surgical procedure. Tumor size and median household income were treated as continuous variables and modeled as a natural cubic spline. Caliper matching was performed by matching participants who were within 0.2 times the standard deviation of their propensity score.^28^ A standardized difference >0.1 was considered a meaningful imbalance between comparison groups.^29^

We calculated hazard ratios (HRs) using the Cox proportional hazards model in SAS, version 9.4 (SAS Institute). For all HRs, 95% confidence limits were generated. *p* values were two‐tailed with a level of significance set at *p* < 0.05.

## 3 RESULTS

There were 10,834 Filipino women and 414,618 White women with breast cancer included in the study. Patient, tumor and treatment characteristics are presented in Table 1. Filipino women were younger on average at diagnosis than White women (57.5 vs. 60.8 years; *p* < 0.0001). On average, Filipino women presented at a higher clinical stage compared with White women (*p* < 0.0001) but the absolute difference was small; 42.4% of Filipino versus 47.1% of White women presented with Stage I disease and 16.4% of Filipino versus 15.4% of White women presented with Stage III–IV disease. Fewer Filipino women had low‐grade tumors than White women (15.3% vs. 21.2% *p* < 0.0001). Filipino women had a greater percentage of tumors over 2 cm in size compared with White women and a greater frequency of lymph node involvement. A greater percentage of Filipino women than White women had HER2‐positive tumors (12.1% vs. 7.4%; *p* < 0.0001). The proportions of ER‐positive and PR‐positive tumors were similar.

**TABLE 1:** Characteristics of Filipino and White women with breast cancer (all stages).

Variable	Filipino	White	*p*‐Value
No. of patients	10,834 (2.5%)	414,618 (97.5%)	
Year of diagnosis			
2004–2006	2188 (20.2%)	95,731 (23.1%)	<0.0001
2007–2009	2544 (23.5%)	102,923 (24.8%)	
2010–2012	2821 (26.0%)	105,512 (25.4%)	
2013–2015	3281 (30.3%)	110,452 (26.6%)	
Age at diagnosis, mean (SD)	57.5 (12.1)	60.8 (13.6)	<0.0001
Marital status			<0.0001
Married	6933 (64.0%)	238,013 (57.4%)	
Widowed	1163 (10.7%)	58,351 (14.1%)	
Never married	1447 (13.4%)	51,471 (12.4%)	
Divorced	859 (7.9%)	47,468 (11.4%)	
Unknown	432 (4.0%)	19,315 (4.7%)	
Annual income, mean (SD)	51,102 (10,351)	47,548 (11,507)	<0.0001
Clinical stage			<0.0001
I	4592 (42.4%)	195,081 (47.1%)	
II	4091 (37.8%)	141,246 (34.1%)	
III	1366 (12.6%)	47,834 (11.5%)	
IV	438 (4.0%)	16,332 (3.9%)	
Unknown	347 (3.2%)	14,125 (3.4%)	
Tumor grade			<0.0001
I	1661 (15.3%)	87,874 (21.2%)	
II	4682 (43.2%)	176,362 (42.5%)	
III	4003 (36.9%)	129,980 (31.3%)	
Unknown	488 (4.5%)	20,402 (4.9%)	
Tumor size, cm (mean, SD)	2.5 (2.1)	2.3 (2.2)	<0.0001
<1 cm	1861 (17.2%)	79,619 (19.2%)	<0.0001
1–2 cm	3177 (29.3%)	144,688 (34.9%)	
2–3 cm	2339 (21.6%)	81,269 (19.6%)	
3–5 cm	1905 (17.6%)	57,174 (13.8%)	
5+ cm	1067 (9.8%)	34,816 (8.4%)	
Unknown	485 (4.5%)	17,052 (4.1%)	
Nodal status (N stage)			0.0002
N0	6882 (63.5%)	271,090 (65.4%)	
N1	2672 (24.7%)	97,412 (23.5%)	
N2	696 (6.4%)	24,162 (5.8%)	
N3	401 (3.7%)	14,176 (3.4%)	
Unknown	183 (1.7%)	7778 (1.9%)	
ER status			<0.0001
Positive	8612 (79.5%)	331,375 (79.9%)	
Negative	1919 (17.7%)	67,844 (16.4%)	
Unknown	303 (2.8%)	15,399 (3.7%)	
PR status			<0.0001
Positive	7311 (67.5%)	285,804 (68.9%)	
Negative	3098 (28.6%)	110,075 (26.5%)	
Unknown	425 (3.9%)	18,739 (4.5%)	
HER2 status			<0.0001
Positive	1314 (12.1%)	30,771 (7.4%)	
Negative	4422 (40.8%)	172,366 (41.6%)	
Unknown	366 (3.4%)	12,827 (3.1%)	
NA	4732 (43.7%)	198,654 (47.9%)	
Surgery			<0.0001
Lumpectomy	4739 (43.7%)	226,450 (54.6%)	
Mastectomy	5363 (49.5%)	160,241 (38.6%)	
None	653 (6.0%)	25,832 (6.2%)	
Unknown	79 (0.7%)	2095 (0.5%)	
Radiotherapy			<0.0001
No	5558 (51.3%)	189,909 (45.8%)	
Yes	4964 (45.8%)	214,038 (51.6%)	
Unknown	312 (2.9%)	10,671 (2.6%)	
Chemotherapy			<0.0001
No/unknown	5475 (50.5%)	243,635 (58.8%)	
Yes	5359 (49.5%)	170,983 (41.2%)	
Mean follow‐up time, years (SD)	5.5 (3.4)	5.7 (3.5)	<0.0001
Vital status			<0.0001
Alive	9381 (86.6%)	333,250 (80.4%)	
Died of breast cancer	853 (7.9%)	40,238 (9.7%)	
Died of other cancer	146 (1.3%)	8440 (2.0%)	
Died of heart diseases	171 (1.6%)	13,103 (3.2%)	
Died of other diseases	117 (1.1%)	11,230 (2.7%)	
Unknown death cause	166 (1.5%)	8357 (2.0%)	

*Note*: Values are presented as *n* (%), unless otherwise specified.

Abbreviations: ER, estrogen receptor; PR, progesterone receptor; SD, standard deviation.

There were some differences regarding treatments received. A greater proportion of Filipino than White women were treated with mastectomy (49.5% vs. 38.6%; *p* < 0.0001). Among women having lumpectomy, a similar proportion of White women received radiotherapy as Filipino women (74.3% vs. 74.5%). Among women having mastectomy, a similar proportion of Filipino women received radiotherapy as White women (25.8% vs. 24.4%). A higher proportion of Filipino women received chemotherapy than White women (49.5% vs. 41.2%; *p* < 0.0001).

**FIGURE 2:**
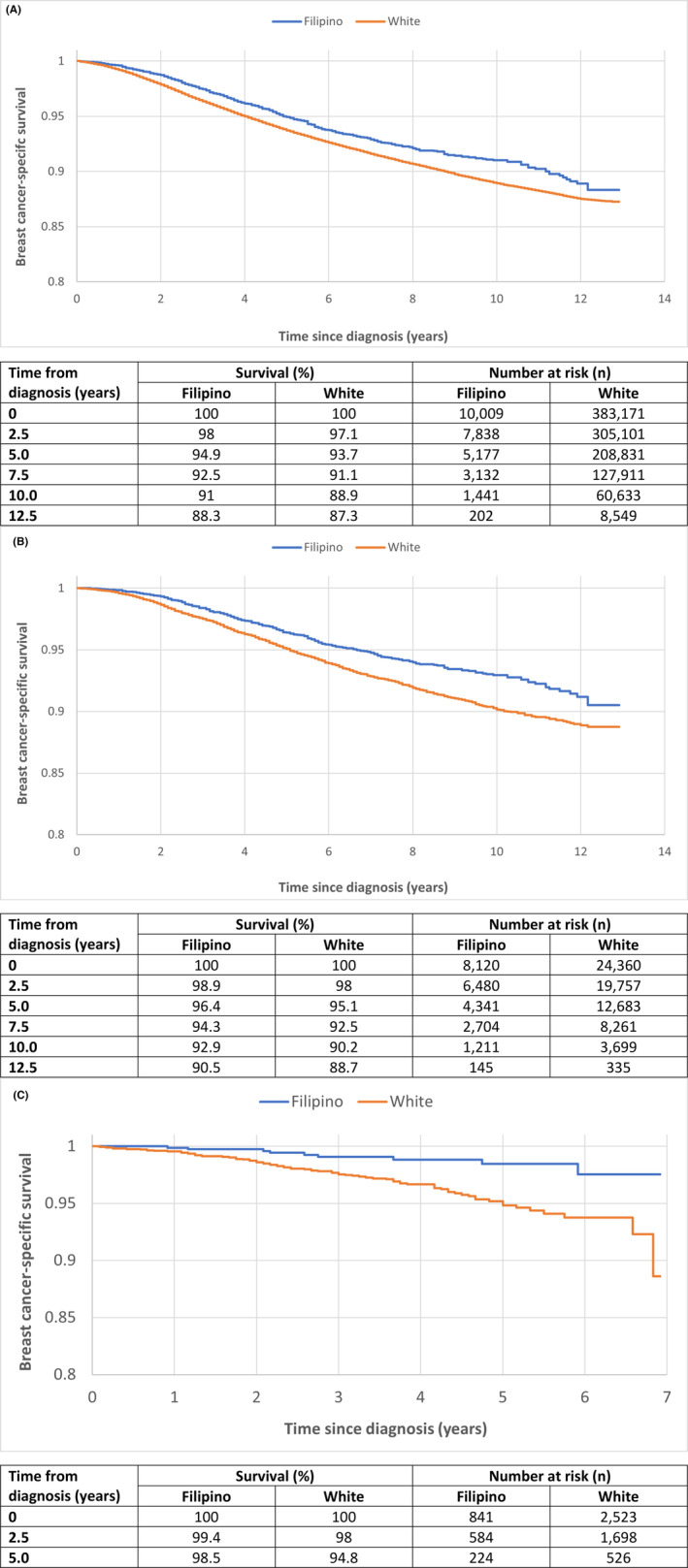
(A) Crude and (B) propensity‐matched Kaplan–Meier survival curves for Filipino versus White women with stage I–III breast cancer. (C) Propensity‐matched Kaplan–Meier survival curves of Filipino versus white women with stage I–III HER2‐positive breast cancers.

**FIGURE 3:**
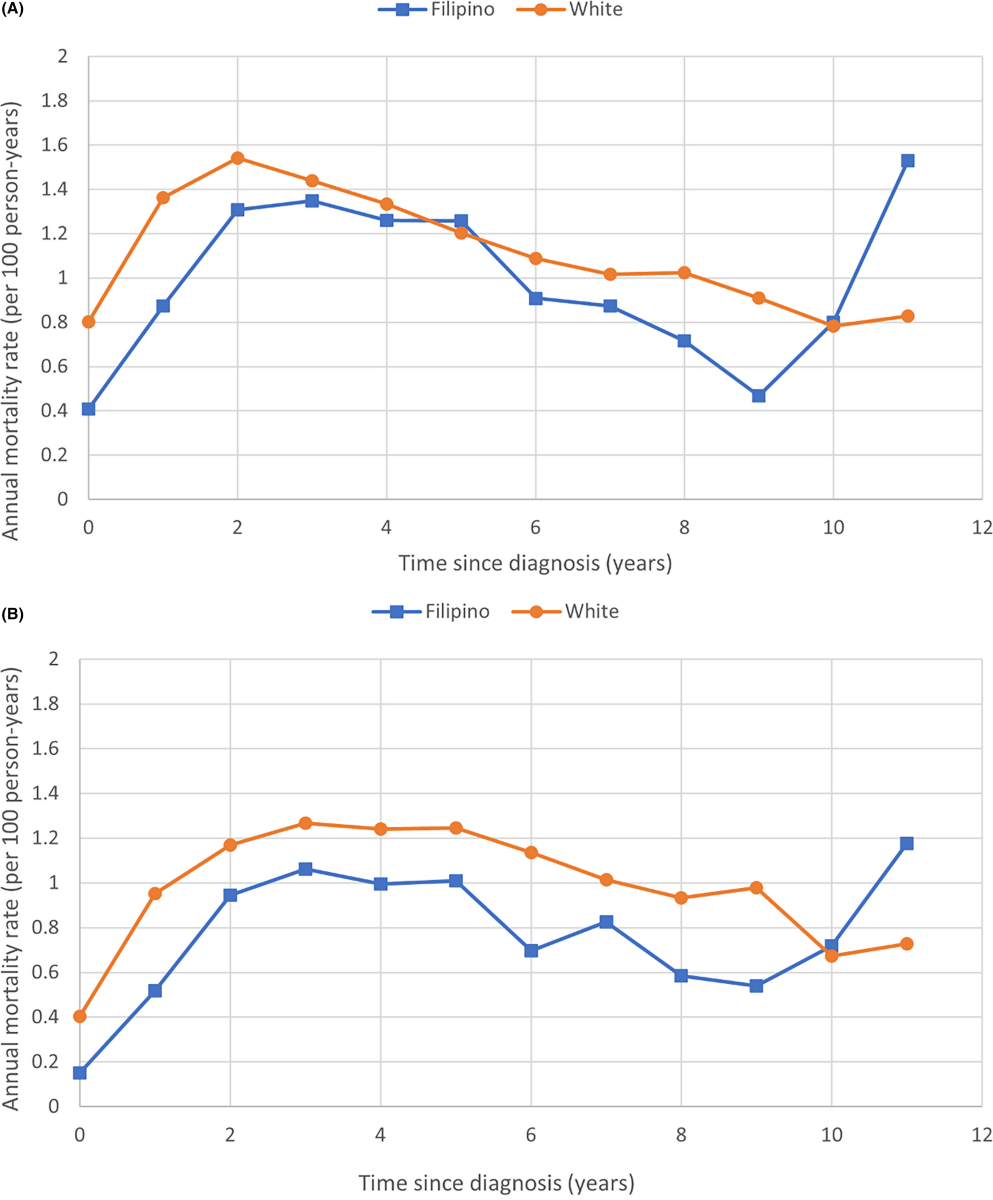
(A) Crude and (B) adjusted annual breast cancer‐specific mortality rates for Filipino versus White women with stage I–III breast cancer.

Among all women with stage I–III breast cancer, the crude 10‐year breast cancer‐specific survival was 91.0% for Filipino women and 88.9% for White women (HR 0.81, 95% CI 0.74–0.88, *p* < 0.0001; Figure 2A). Over the 10‐year period, the annual mortality rate was 1.2% for White women and 0.9% for Filipino women. In the first 9 years following diagnosis, annual mortality rates in White women exceeded those in Filipino women (Figure 3A).

In our propensity‐matched analysis, we matched 8120 Filipino women with 24,360 White women (1:3 matching). After matching, the two groups were similar for age and year of diagnosis, marital status, clinical stage, grade, size and nodal status, receptor status and breast surgical procedure (Table S1). The cancer‐specific survival from breast cancer after 10 years of follow‐up was 92.9% for Filipino women and 90.2% for White women (HR = 0.73, 95% CI 0.66–0.81, *p* < 0.0001; Figure 2B). Filipino women had better survival than White women within subgroups defined by age of diagnosis, tumor grade, stage, nodal status, and receptor status (Table 2). A pronounced survival advantage was seen in Filipino patients with HER2‐positive disease (HR 0.38, 95% CI 0.20–0.71, *p* = 0.002; Figure 2C). Other subgroups of note included node‐negative (HR 0.68, 95% CI 0.57–0.82) and Stage II disease (HR 0.68, 95% CI 0.58–0.79). Annual mortality rates in the matched analysis followed a similar pattern as with the unmatched analysis (Figure 3B).

**TABLE 2:** Adjusted hazard ratios for breast cancer–specific death in Filipino versus White women with Stage I‐IIIC breast cancer (matched analysis).

Parameter	Hazard ratio (95% CI)	*p*‐Value
Overall	0.729 (0.656–0.810)	<0.0001
Age at diagnosis, years		
≤50	0.776 (0.647–0.931)	0.006
>50	0.707 (0.621–0.805)	<0.0001
Tumor grade		
I/II	0.710 (0.593–0.851)	0.0002
III	0.739 (0.649–0.842)	<0.0001
Stage		
I	0.782 (0.601–1.016)	0.07
II	0.679 (0.583–0.790)	<0.0001
III	0.788 (0.660–0.939)	0.008
Nodal status		
N0	0.683 (0.572–0.816)	<0.0001
N1‐N3	0.757 (0.664–0.863)	<0.0001
Estrogen receptor status		
Positive	0.708 (0.622–0.805)	<0.0001
Negative	0.776 (0.645–0.933)	0.007
HER2 receptor status		
Positive	0.377 (0.201–0.708)	0.002
Negative	0.650 (0.515–0.823)	0.0003

In Table S2, we compared the HRs for various prognostic factors separately in Filipino and White women with Stage I‐III breast cancer. Age at diagnosis and tumor factors predicted breast cancer‐specific death in both subgroups. Age > 60 years (compared with 50–59 years), tumor size >2 cm (compared with 1–1.9 cm), grade, lymph node burden, and ER negativity predicted worse survival. HER2 positivity was associated with better survival.

## 4 DISCUSSION

In this population‐based cohort study of breast cancer patients in the USA, Filipino women had a significantly better 10‐year actuarial breast cancer‐specific survival than White women. Over a 10‐year follow‐up period, Filipino women with Stage I–IIIC breast experienced a 27% lower rate of death compared with a matched cohort of White women with similar cancers (HR = 0.73).

There is a paucity of knowledge regarding breast cancer characteristics and survival outcomes among the various Asian groups because Asian women are often studied as a whole.^15^, ^16^, ^30^, ^31^ When individual Asian ethnic groups are studied, clinically significant differences in presentation, tumor characteristics and clinical outcomes may be appreciated.^14^, ^17^, ^18^, ^32^, ^33^, ^34^, ^35^, ^36^

We observed that Filipino women were younger on average at diagnosis and had more advanced breast cancers (larger size, higher grade, more positive lymph nodes, and were more likely to be HER2‐positive). Our results are consistent with the findings by Simpson et al.^22^ at the institutional level. These findings reinforce the importance of early detection and increasing awareness in women of Filipino ancestry. The recent update from the United States Preventive Services Task Force on starting breast cancer screening at age 40 may help detect cancers earlier in Filipino women.^37^

There are known racial differences in breast cancer molecular subtypes. Compared with White women, Black women have a higher frequency of triple‐negative or basal‐type breast cancer, whereas Asians (including Chinese and Filipino women) have a higher prevalence of HER2‐positive breast cancer.^17^, ^18^, ^38^, ^39^ In our study, Filipino women had a higher prevalence of HER2‐positive cancer but were similar to White women for ER and PR status. After matching for size, grade, nodal status, and ER/PR/HER2 status, the HR for breast cancer death was 0.73 for Filipino versus White women. Differences in tumor genomics may account for some of the disparity, as there may be differences in tumor biology related to genetic ancestry that are not captured by standard measures. While there have been limited reports of differences in genomic mutations in breast cancers from Chinese or Asian versus White women (i.e., increased prevalence of *TP53* and *AKT1* mutations),^40^, ^41^ we are not aware of any such studies in Filipino women. Among women with West African ancestry, the Duffy‐Null allele is independently associated with an increased risk of triple negative breast cancer.^42^, ^43^ In the prospective, randomized TAILORx trial assessing the benefit of chemotherapy among 9396 women with ER/PR‐positive, HER2‐negative, node‐negative breast cancer, despite comparable 21‐gene recurrence score distributions, Black (3.9%), Asian (3.6%) and Hispanic (3.1%) women had higher 8‐year locoregional recurrence rates compared with White women (1.8%, *p* < 0.001).^44^ However, compared with White women (relapse‐free interval, RFI 92.3%, overall survival, OS 93.4%), Black women had higher 9‐year rates of recurrence (89.1%) and worse overall survival (89.9%), while Asian (RFI 92.1%, OS 96.9%) women had better oncologic outcomes.^45^ Together, these studies suggest that tumor genomics may underlie some breast cancer disparities.^46^

Despite the higher prevalence of negative prognostic factors, we observed a better crude survival rate among Filipino women and when we matched for all prognostic factors, the gap in survival persisted. A greater proportion of Filipino women were married and had higher annual household income compared with White women, which suggests better socioeconomic status and possibly access to timely care.^47^, ^48^ Borrell et al. have highlighted that race and ethnicity are sociopolitical constructs that serve as proxies for complex social structures that improve or inhibit access to care, which likely are not entirely captured in the SEER database.^49^ While minority groups face barriers to care, Filipinos in the United States represent individuals who are relatively wealthy and have higher rates of college education compared with other immigrant populations. Filipino Americans also have lower poverty rates, have health insurance coverage, and are more likely to be covered by private health insurance compared with other foreign‐born or US‐born populations.^50^ Filipino Americans also have the highest rates of English proficiency compared with other Asian American subgroups.^51^ Filipino Americans also have unique considerations, as groups with older immigration histories, including Filipino Americans, have higher incidence of cancers more commonly found in the United States, including breast and colorectal cancer, than other Asian immigrant subgroups.^26^ Despite our data suggesting better survival for Filipina American women with breast cancer compared with White women, breast cancer is the leading cause of cancer death among Filipina American women.^34^

We expect that the increased number of Filipino women receiving chemotherapy relates to the relatively younger age of diagnosis, more advanced tumors, and more HER2‐positivity.^52^ However, in the matched analysis, we adjusted for age at diagnosis and the difference in survival was still apparent. There may be differences in adherence and compliance with treatment, which are not captured in SEER. Information on endocrine therapy was not available. Nevertheless, in both the ER‐positive and HER2‐positive tumor subgroups, Filipino women had better survival than White women. The difference in survival among HER2‐positive women was most striking (Figure 2C). There may be a difference in treatment adherence or differences in the metabolism of systemic treatment between White and Asian women (e.g., pharmacogenetics).^53^

Previous work comparing the survival of Filipino and White women in the SEER database revealed conflicting findings.^13^, ^54^, ^55^ In contrast to these prior studies, our study analyzed a large contemporary cohort, we had a longer follow‐up period, and a specific focus on one Asian ethnic group.^17^, ^18^ We compared Filipino and White women with breast cancer in the same healthcare system and used propensity matching, a modern approach to analyzing observational data.

To gain more insight on the differences in breast cancer mortality, we reviewed both cancer incidence and case‐fatality rates. From 2003 to 2011, breast cancer mortality in the United States was 15.2, 9.9 and 23.3 per 100,000 for Filipino, Chinese and White women, respectively (adjusted to the 2000 US census population).^33^ Age‐adjusted breast cancer incidence in the United States from 2009 to 2011 was 111.3, 82.8, and 134.4 per 100,000 for Filipino, Chinese, and White women (adjusted to the 2000 US census population).^56^ In our study, the actuarial mortality rate from breast cancer at 10 years was 7.1% for Filipino women and 9.8% for White women.

In 2020, according to Globocan, breast cancer mortality was 19.3, 10.0, and 12.4 per 100,000 for Philippines, China, and the United States, respectively, while the incidence was 52.7, 39.1, and 90.3 per 100,000 for Philippines, China, and the United States, respectively.^2^ If these data are accurate, then Filipino women in the United States have much lower mortality rates than Filipino women in the Philippines (15.2 vs. 19.3 per 100,000 per year) which could be attributed to a much lower case fatality (Table 3). Filipino women living in the Philippines are reported to have much worse 5‐year survival than Filipino women living in the US (49.5% vs. 82.2%, *p* < 0.001).^54^, ^58^ The disparity in survival may be due to late stage at presentation, inadequate screening^59^ and access to cancer care. Ho et al. recently characterized the significant challenges of women accessing breast and cervical cancer screening in the Philippines, highlighting high out‐of‐pocket costs, centralization of health resources and infrastructure in the capital, lack of organized screening programs, lower health literacy and gendered sociocultural pressures as barriers.^60^ Co et al. have reported that challenges to accessing surgical care for breast cancer in the Philippines include socioeconomic and geographic disparities and the relative low number of surgeons.^61^

**TABLE 3:** Comparison of incidence, mortality, and case‐fatality rates between Filipino, Chinese and White women with breast cancer.

Population Location	Mortality (per 100,000/year [ref])	Incidence (per 100,000/year [ref])	Case Fatality (% of case patients who died of breast cancer [ref])
Philippines	19.3^a^ (2020)^1^	52.7^a^ (2020)^1^	50.5^a^ (1993–2002)^54^
Filipina in United States	15.2^b^ (2003–2011)^33^	111.3^b^ (2009–2011)^56^	7.1^c^ (2004–2015)
White in United States	23.3^b^ (2003–2011)^33^ 12.4^a^ (2020)^3^	134.4^b^ (2009–2011)^56^ 90.3^a^ (2020)^3^	9.8^c^ (2004–2015)
Chinese in United States	9.9^b^ (2003–2011)^33^	82.8^b^ (2009–2011)^56^	13.9 (2004–2015)^19^
China	10.0^a^ (2020)^2^	39.1^a^ (2020)^2^	18.0 (1997–2001^d^)^57^

Abbreviation: ref, reference number.

^a^ Age‐adjusted to the world standard population

^b^ Age‐adjusted to the 2000 US census population

^c^ Current study

^d^ Vital status followed until 2007.

The difference in incidence many be explained by increased cancer screening and early detection in the United States compared to the Philippines.^62^ While some individuals may benefit from early cancer screening, others may be diagnosed with cancers that are clinically insignificant (i.e., overdiagnosis bias).^63^ Thus, the incidence rate is increased but the case‐fatality rate is effectively reduced from overdiagnosis. There may disparities in cancer screening faced by Filipina American women with cancer, as the incidence of localized and distant disease are increasing significantly, particularly in young Filipina Americans.^35^ Mammography screening rates in California are slightly lower in Asian American women than in non‐Hispanic White, non‐Hispanic Black, and Hispanic women^35^ but among Asian American women, Filipina Americans do have the highest rates of mammography screening.^64^ Among Filipina Americans, unmarried and uninsured women were less likely to receive mammograms than married and insured women,^65^ revealing potential socioeconomic barriers to cancer screening. In comparison to other Asian American women, Filipina women were less likely to be insured and use primary care,^66^ both of which are predictors of receiving mammograms among Asian American subgroups.^67^, ^68^ Oviedo has reported that mammography reminders from a healthcare provider improves mammogram adherence among Filipino American women, highlighting the importance of the primary care provider.^69^ Interestingly, McMenamin and colleagues found that privately insured Asians still had lower mammography rates than non‐Hispanic White women (83.1 vs. 87.6%), suggesting that providing insurance coverage alone has limitations without addressing cultural factors.^70^ In a small qualitative study of 12 Filipino women, perceived barriers to screening mammography and clinical breast examination include fear, lack of finances and difficulty finding time to receive care.^59^ Other barriers to screening include different mindsets and healthcare systems in the Philippines with respect to early detection, unpleasant mammography experiences, difficulty accessing services, and cultural beliefs.^71^

Finally, our data highlights the importance of disaggregating the Asian American subgroups when examining health statistics, in order to truly identify disparities in health outcomes among Asian Americans. The better survival of Filipina American women with breast cancer plays into the “model minority myth” that Asian Americans are models of good minority health due to their educational success, financial achievements, and overcoming barriers and challenges.^51^ As a result, healthcare providers' assumption of the model minority myth may lead to neglect of screening and healthy lifestyle recommendations.^51^ We highlight that not all Asian American women with breast cancer experience as good survival outcomes as Filipina Americans and aggregating health data among the Asian American subgroups masks these clinically relevant within‐group differences.^72^

There are several limitations to our study. While SEER data on chemotherapy and radiation therapy initiation was included, it lacked specific details including treatment type, dose, duration, and adherence.^23^ We were unable to discern Filipino women born in the United States from those born in the Philippines, which would be important given that immigration status has substantial influence on breast cancer survival.^7^

In summary, 10‐year breast cancer‐specific survival is superior among Filipino women compared with White women with breast cancer in the SEER database. We observed a 27% decreased rate of breast cancer mortality among Filipino compared with White women. The difference in breast cancer–specific survival between Filipino and White women may be a result of genomic tumor differences that remain to be elucidated and importantly race‐dependent socioeconomic disparities in access to care. The difference between breast cancer mortality rates among Filipino women with breast cancer in the USA versus the Philippines needs to be explored. Efforts should be aimed at promoting screening in young Filipino women in order to detect breast cancers at an earlier stage.

## AUTHOR CONTRIBUTIONS

**David W. Lim:** Conceptualization (supporting); data curation (equal); formal analysis (equal); investigation (equal); methodology (supporting); writing – original draft (equal); writing – review and editing (lead). **Winston W. Li:** Formal analysis (supporting); visualization (lead); writing – original draft (equal); writing – review and editing (equal). **Vasily Giannakeas:** Conceptualization (equal); data curation (lead); formal analysis (lead); visualization (equal). **Tulin D. Cil:** Conceptualization (equal); methodology (supporting); supervision (supporting); writing – review and editing (equal). **Steven A. Narod:** Conceptualization (lead); formal analysis (supporting); funding acquisition (lead); methodology (lead); resources (lead); software (lead); supervision (lead); writing – review and editing (equal).

## FUNDING INFORMATION

This work was supported by the Peter Gilgan Center for Research on Women's Cancers at Women's College Hospital. V.G. is supported by a Canadian Institutes of Health Research (CIHR) Frederick Banting & Charles Best Canada Graduate Scholarship – Doctoral Research Award. D.W.L. is supported by the CIHR Fellowship and the Canadian Cancer Society Chair in Breast Cancer Research at Women's College Research Institute (Women's College Hospital, Toronto, Ontario, Canada). D.W.L. is also the recipient of a 2021 Conquer Cancer Young Investigator Award for Invasive Lobular Carcinoma Research supported by Conquer Cancer, the ASCO Foundation, and the Lobular Breast Cancer Alliance. T.D.C. is supported by the Gattuso Chair in Breast Surgical Oncology at University Health Network (Toronto, Ontario, Canada). S.A.N. is supported by a Tier 1 Canada Research Chair in Breast Cancer.

## CONFLICT OF INTEREST STATEMENT

The authors have no conflict of interest to declare.

## Supporting information


Table S1.

Table S2.

## Data Availability

The data that support the findings of this study are available from the National Cancer Institute Surveillance, Epidemiology, and End Results Program. Restrictions apply to the availability of these data, which were used under license for this study. Data are available at https://seer.cancer.gov/data/access.html with the permission of the Surveillance Research Program (SRP) in the National Cancer Institute's Division of Cancer Control and Population Sciences (DCCPS).
