# Supplementary material for: Survival of Filipino women with breast cancer in the United States
Source: Cancer Med. 2023 Sep 27;12(19):19921–34. doi: 10.1002/cam4.6403 (PMC10587940; doi:10.1002/cam4.6403)
Supplement: Supplementary file 1 — Table S1. [file CAM4-12-19921-s001.docx]

| **Supplemental TABLE 1. Characteristics of Filipino and White Women with Stage I-IIIC Breast Cancer (Matched Analysis)** | | | |
| --- | --- | --- | --- |
| **Variable** | **Filipino** | **White** | **P-Value** |
| No. of patients | 8,120 (25.0%) | 24,360 (75.0%) |  |
| Year of diagnosis |  |  | 0.9 |
| 2004-2006 | 1,676 (20.6%) | 4,991 (20.5%) |  |
| 2007-2009 | 1,972 (24.3%) | 5,953 (24.4%) |  |
| 2010-2012 | 2,068 (25.5%) | 6,129 (25.2%) |  |
| 2013-2015 | 2,404 (29.6%) | 7,287 (29.9%) |  |
| Age at diagnosis, years (mean, SD) | 57.5 (11.6) | 57.5 (11.5) | 1 |
| Marital status |  |  | 0.06 |
| Married | 5,333 (65.7%) | 16,091 (66.1%) |  |
| Never married | 1,024 (12.6%) | 3,226 (13.2%) |  |
| Widowed | 817 (10.1%) | 2,268 (9.3%) |  |
| Divorced | 662 (8.2%) | 1,858 (7.6%) |  |
| Unknown | 284 (3.5%) | 917 (3.8%) |  |
| Annual income, USD (mean, SD) | 50,871 (9,915) | 51,281 (10,187) | 0.002 |
| Clinical Stage |  |  | 1 |
| I | 3,939 (48.5%) | 11,817 (48.5%) |  |
| II | 3,454 (42.5%) | 10,362 (42.5%) |  |
| III | 727 (9.0%) | 2,181 (9.0%) |  |
| Tumor Grade |  |  | 1 |
| I | 1,414 (17.4%) | 4,242 (17.4%) |  |
| II | 3,786 (46.6%) | 11,358 (46.6%) |  |
| III | 2,920 (36.0%) | 8,760 (36.0%) |  |
| Tumor Size, cm (mean, SD) | 2.1 (1.8) | 2.1 (1.7) | 0.9 |
| Nodal status (N stage) |  |  | 1 |
| N0 | 5,654 (69.6%) | 16,962 (69.6%) |  |
| N1 | 1,904 (23.4%) | 5,712 (23.4%) |  |
| N2 | 417 (5.1%) | 1,251 (5.1%) |  |
| N3 | 145 (1.8%) | 435 (1.8%) |  |
| ER status |  |  | 1 |
| Positive | 6,872 (84.6%) | 20,616 (84.6%) |  |
| Negative | 1,248 (15.4%) | 3,744 (15.4%) |  |
| PR status |  |  | 0.4 |
| Positive | 5,878 (72.4%) | 17,616 (72.3%) |  |
| Negative | 2,143 (26.4%) | 6,487 (26.6%) |  |
| Unknown | 99 (1.2%) | 257 (1.1%) |  |
| HER2 status |  |  | 1 |
| Positive | 841 (10.4%) | 2,523 (10.4%) |  |
| Negative | 3,631 (44.7%) | 10,893 (44.7%) |  |
| Not Available | 3,648 (44.9%) | 10,944 (44.9%) |  |
| Surgery |  |  | 1 |
| Lumpectomy | 4,131 (50.9%) | 12,394 (50.9%) |  |
| Mastectomy | 3,989 (49.1%) | 11,966 (49.1%) |  |
| Radiotherapy |  |  | 0.03 |
| No | 3,895 (48.0%) | 11,267 (46.3%) |  |
| Yes | 3,984 (49.1%) | 12,367 (50.8%) |  |
| Unknown | 241 (3.0%) | 726 (3.0%) |  |
| Chemotherapy |  |  | 0.004 |
| No/Unknown | 4,172 (51.4%) | 12,970 (53.2%) |  |
| Yes | 3,948 (48.6%) | 11,390 (46.8%) |  |
| Mean follow-up time (years, SD) | 5.8 (3.4) | 5.8 (3.4) | 0.6 |
| Vital status |  |  | <.0001 |
| Alive | 7,399 (91.1%) | 21,584 (88.6%) |  |
| Died of breast cancer | 337 (4.2%) | 1,399 (5.7%) |  |
| Died of other cancer | 98 (1.2%) | 346 (1.4%) |  |
| Died of heart diseases | 106 (1.3%) | 362 (1.5%) |  |
| Died of other diseases | 81 (1.0%) | 402 (1.7%) |  |
| Unknown death cause | 99 (1.2%) | 267 (1.1%) |  |

Values are presented as n (%), unless otherwise specified.

*cm* centimetre, *ER* estrogen receptor, *PR* progesterone receptor, *SD* standard deviation
