# Supplementary material for: Survival of Filipino women with breast cancer in the United States
Source: Cancer Med. 2023 Sep 27;12(19):19921–34. doi: 10.1002/cam4.6403 (PMC10587940; doi:10.1002/cam4.6403)
Supplement: Supplementary file 2 — Table S2. [file CAM4-12-19921-s002.docx]

| SUPPLEMENTAL TABLE 2. Risk of Breast Cancer-Specific Death amongst (A) Filipino and (B) white women with stage I-III breast cancer | | | | |
| --- | --- | --- | --- | --- |
| **(A)  Filipino women** |  |  |  |  |
|  | Unadjusted | | Adjusted | |
|  | **HRs (95% CI)** | **P-value** | **HRs (95% CI)** | **P-value** |
| Age (years) |  |  |  |  |
| <40 | 1.862 (1.377-2.517) | < .0001 | 1.304 (0.953-1.786) | 0.1 |
| 40-50 | 0.956 (0.749-1.221) | 0.7 | 0.845 (0.658-1.084) | 0.2 |
| 50-60 | Ref |  | Ref |  |
| 60+ | 1.149 (0.937-1.409) | 0.2 | 1.33 (1.078-1.641) | 0.008 |
| Tumour Size (cm) |  |  |  |  |
| <1 |  |  |  |  |
| 1-1.9 | 0.4 (0.236-0.677) | 0.006 | 0.508 (0.299-0.863) | 0.01 |
| 2-2.9 | Ref |  | Ref |  |
| 3-4.9 | 2.628 (1.975-3.498) | < .0001 | 1.878 (1.399-2.523) | < .0001 |
| 5+ | 4.209 (3.189-5.555) | < .0001 | 2.31 (1.719-3.104) | < .0001 |
|  | 9.939 (7.515-13.145) | < .0001 | 4.295 (3.14-5.874) | < .0001 |
| Nodal Status |  |  |  |  |
| N0 | Ref |  | Ref |  |
| N1 | 3.232 (2.631-3.969) | < .0001 | 2.165 (1.736-2.699) | < .0001 |
| N2 | 6.755 (5.278-8.644) | < .0001 | 3.4 (2.591-4.461) | < .0001 |
| N3 | 11.773 (9.009-15.385) | < .0001 | 4.972 (3.656-6.763) | < .0001 |
| ER Status |  |  |  |  |
| Positive | Ref |  | Ref |  |
| Negative | 2.439 (2.034-2.924) | < .0001 | 1.733 (1.411-2.129) | < .0001 |
| HER2 Status |  |  |  |  |
| Positive | 1.07 (0.727-1.574) | 0.7 | 0.614 (0.412-0.915) | 0.0166 |
| Negative | Ref |  | Ref |  |
| Tumour grade |  |  |  |  |
| 1 | Ref |  | Ref |  |
| 2 | 4.604 (2.622-8.086) | < .0001 | 3.039 (1.68-5.499) | 0.0002 |
| 3 | 10.838 (6.232-18.849) | < .0001 | 4.562 (2.507-8.302) | < .0001 |
|  |  |  |  |  |
| **(B) White women** | |  |  |  |
|  | Unadjusted |  | Adjusted |  |
|  | **HRs (95% CI)** | **P-value** | **HRs (95% CI)** | **P-value** |
| Age (years) |  |  |  |  |
| <40 | 1.729 (1.644-1.818) | < .0001 | 1.078 (1.023-1.135) | 0.005 |
| 40-50 | 1.016 (0.978-1.056) | 0.4 | 0.913 (0.878-0.95) | < .0001 |
| 50-60 | Ref |  | Ref |  |
| 60+ | 1.265 (1.228-1.304) | < .0001 | 1.61 (1.561-1.66) | < 0.0001 |
| Tumour Size (cm) |  |  |  |  |
| <1 |  |  |  |  |
| 1-1.9 | 0.439 (0.413-0.467) | < .0001 | 0.56 (0.526-0.596) | < 0.001 |
| 2-2.9 | Ref |  | Ref |  |
| 3-4.9 | 2.393 (2.307-2.482) | < .0001 | 1.748 (1.683-1.815) | < .0001 |
| 5+ | 4.373 (4.22-4.531) | < .0001 | 2.583 (2.486-2.685) | < .0001 |
|  | 7.215 (6.951-7.489) | < .0001 | 3.674 (3.524-3.831) | < .0001 |
| Nodal Status |  |  |  |  |
| N0 | Ref |  | Ref |  |
| N1 | 2.721 (2.644-2.8) | < .0001 | 1.884 (1.827-1.944) | < .0001 |
| N2 | 5.567 (5.372-5.768) | < .0001 | 2.979 (2.864-3.099) | < .0001 |
| N3 | 10.44 (10.051-10.844) | < .0001 | 4.856 (4.651-5.07) | < .0001 |
| ER Status |  |  |  |  |
| Positive | Ref |  | Ref |  |
| Negative | 2.739 (2.668-2.811) | < .0001 | 1.83 (1.775-1.886) | < .0001 |
| HER2 Status |  |  |  |  |
| Positive | 1.25 (1.179-1.325) | < .0001 | 0.732 (0.689-0.777) | < .0001 |
| Negative | Ref |  | Ref |  |
| Tumour grade |  |  |  |  |
| 1 | Ref |  | Ref |  |
| 2 | 2.698 (2.562-2.842) | < .0001 | 1.792 (1.701-1.888) | < .0001 |
| 3 | 6.502 (6.184-6.836) | < .0001 | 2.802 (2.655-2.957) | < .0001 |
